# Experimental Study on Axial Compressive Performance of Polyvinyl Alcohol Fibers Reinforced Fly Ash—Slag Geopolymer Composites

**DOI:** 10.3390/polym14010142

**Published:** 2021-12-30

**Authors:** Shuhua Xiao, Yongjian Cai, Yongchang Guo, Jiaxiang Lin, Guotao Liu, Xuewei Lan, Ying Song

**Affiliations:** 1 School of Civil and Transportation Engineering, Guangdong University of Technology, Guangzhou 510006, China; 2111709023@mail2.gdut.edu.cn (S.X.); CYJ_GDUT@163.com (Y.C.); hz.xzh@163.com (Y.S.); 2 Guangdong GW Metal Industry Group Co., Ltd., Guangzhou 510030, China; gwjsim@gwjscyjt.com; 3 Guangzhou Zengcheng Zhengyuan Construction Engineering Testing Center Co., Ltd., Guangzhou 511300, China; zyjc06@163.com

**Keywords:** polyvinyl alcohol (PVA) fiber, low-calcium fly ash, slag, scanning electron microscope (SEM), fiber reinforced geopolymer concrete (FRGC)

## Abstract

Geopolymer concrete (GC) has been gaining attention in research and engineering circles; however, it is a brittle material with poor tensile performance and crack resistance. To address these problems, we introduced fibers into GC. In this study, axial compression and scanning electron microscope (SEM) tests were carried out on polyvinyl alcohol (PVA) short fiber reinforced low-calcium fly ash-slag-based geopolymer concrete (PFRGC). The ratio of PVA short fibers and low-calcium fly ash on the compression behavior of fiber reinforced geopolymer concrete (FRGC) were investigated and discussed. The test results show that PVA fibers play a bridging role in the cracks of the specimen and bear the load together with the matrix, so the addition of PVA fibers delayed the crack propagation of GC under axial compression. However, with the increase of low-calcium fly ash/PVA fibers, the number of unreacted fly ash particles in PFRGCs increases. Too many unreacted fly ash particles make GC more prone to micro-cracks during loading, adversely affecting compressive properties. Therefore, the axial compressive strength, elastic modulus, and Poisson’s ratio of GC decrease with the increasing low-calcium fly ash/PVA fibers.

## 1. Introduction

Global warming and climate change are mainly caused by carbon dioxide (CO_2_) emissions, which have become the focus of international attention [1,2]. Construction industry emissions are one of the top three sources of greenhouse gases in the world [3,4], which emits 36% of greenhouse gases [5]. In particular, construction activities in developing countries are booming, and their greenhouse gas emissions account for more than half of the global construction industry emissions [6]. According to the report, the production of 1 kg of ordinary Portland cement produces 0.66–0.82 kg of carbon emission [7]. The emission of CO_2_ in the manufacturing process of ordinary Portland cement is mainly due to the calcination of calcium carbonate (CaCO_3_). Compared with ordinary Portland cement, the production of geopolymer can not only fully utilize industrial solid waste as raw materials, but also directly react at room temperature, so the use of geopolymer can reduce 73% of greenhouse-gas emission and 43% of energy consumption [8]. Many scholars try to improve the environment by studying geopolymer concrete (GC) instead of ordinary concrete [9,10,11].

Using GC instead of cement concrete can reduce the problems caused by limestone mining, ground granulated blast furnace slag and fly ash [12]. GC materials can be natural sources, such as metakaolin, or industrial by-products, such as fly ash, GGBS, rice husk ash, and high calcium wood ash [13,14,15]. The synthetic cost of GC is lower than that of ordinary Portland cement concrete (PCC), and its economic index is also lower. According to the research report conducted by Singh [16], the production cost of GC is only about 75% of that of PCC. At high temperatures, the strength loss of PCC is severe, while the residual strength retention rate of fly ash-based GC is higher [17], and the splitting tensile property [18] and spalling resistance are superior to PCC. The chloride ion erosion resistance of GC with well-designed composition is equal to or better than that of PCC. The high alkalinity of the matrix is beneficial to the corrosion resistance of steel bars in concrete [19,20]. However, the properties of GC prepared from different materials are significantly different.

Many studies on GC show that GC has the advantages of acid resistance, low creep, and high compressive strength, but its weakness lies in its brittleness [21,22]. Sarker et al. [23] compared the brittleness of GC and PPC, and found that GC has more suffocating fracture surfaces than PPC. Pan et al. [24] reported that the change of binder material morphology affected the microstructure of the matrix, resulting in the reduction of fracture load. Must reduce this brittleness to achieve high-performance and sustainable building materials.

To improve the ductility and toughness of GC and limit crack growth, many scholars have introduced fibers into GC [25,26,27]. High modulus fibers improve the mechanical properties, while low modulus fibers improve the behavior after cracking. The addition of fibers improves the mechanical properties, structural integrity, ductility, and impact strength of concrete [28,29,30]. PVA fibers have a high elastic modulus of 29–42 GPa and tensile strength of 0.8–2.5 GPa. PVA fiber has been widely used in the industry. PVA fiber has practical applications, such as in clothing and porous materials [31,32]. In addition, due to hydroxyl groups in the molecular chains, PVA fibers have a strong chemical binding with cementing adhesive. Almashhadani et al. [33] confirmed the strong bond formation between PVA and geopolymer. Zhang et al. [34] proved that PVA fibers positively effect geopolymers under freeze–thaw conditions. Li et al. [35] indicated that the flexural strength, ductility, and toughness of geopolymer concrete containing PVA fibers improved. Moreover, PVA fibers reinforced geopolymer concrete has higher impact resistance [36,37]. Although PVA-fiber reinforced geopolymer concrete has been studied, the research on PVA-fiber reinforced geopolymer concrete with different content of aggregate and different amounts of PVA short fibers is limited.

This study used PVA short fibers and low-calcium fly ash to fabricate the FRGC. For convenience, PVA short fibers and low-calcium fly ash were called PVA fibers and fly ash, respectively. Axial compression test and scanning electron microscope (SEM) test were used to study the axial compression performance of PVA fibers reinforced fly ash-slag-based geopolymer concrete (PFRGC) specimens. A total of 30 columns were designed according to the two variables (mass ratio of fly ash and volume ratio of PVA fiber), of which three were identical. The effect of the ratio of fly ash and PVA fibers on the failure mode, compressive strength, the stress–strain behavior, elastic modulus, and Poisson’s ratio were investigated and discussed.

## 2. Experiment Program

### 2.1. Materials

**Figure 1:**
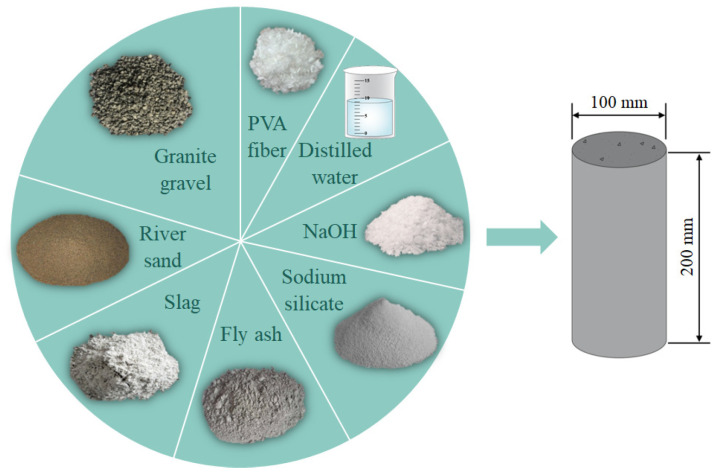
Material composition of PFRGCs.

The PFRGCs are composed of slag, fly ash, sodium hydroxide, sodium silicate powder, river sand, PVA fibers, and water, as shown in Figure 1. The primary raw materials were slag and fly ash to prepare the geopolymer binder. Slag is a highly active raw material, which can significantly improve the early strength and compactness of GC when mixed into silicon-rich aluminum raw materials such as fly ash or volcanic ash. Because of the high content of Ca in slag, a certain amount of calcium aluminosilicate gel (C-(A)-S-H) can be formed when it is mixed into the silicon-rich aluminum raw material system. When the slag content is low, it can coexist with the main alkali-activated product aluminosilicate gel (N-A-S-(H)). However, when the content of Ca is increased to a certain extent (for example, the Ca/Si ratio of cementitious materials is above 0.2 [38]), the product is mainly C-(A)-S-H, and N-A-S-(H) is difficult to coexist. In addition, from the composition of cementitious materials, the higher the Si/Al ratio, the higher the strength [39]. Different sources will also lead to the difference in GC performance for the same kind of raw materials, which is also a difficult point in the standardization of GC mix design. The slag was blast-furnace slag powder with a specific surface area of 430 m^2^/kg produced by Henan Borun Casting Materials Co., Ltd., which belongs to Grade S95 slag. The fly ash produced by Hebei Huihao Environmental Protection Technology Co., Ltd. has a density of 2.4 g/cm^3^ and a specific surface area of 460 m^2^/kg. The detailed chemical composition of the fly ash is shown in Table 1. The sodium hydroxide (NaOH) with a density of 2130 kg/m^3^ used in this experiment is industrial grade flake NaOH produced by the Junzheng Group. The instant sodium silicate powder produced by Gongyi Changlong Paohua Alkali Factory has a fineness modulus of 2.84, a SiO_2_ volume ratio of 58.4%, and a Na_2_O volume ratio of 21.2%. The water used in the preparation of the alkali activator is distilled water. The fine aggregate used in this study is river sand, with a grain size of 0.11–0.25 mm, a density of 2.61 g/cm^3^ and a water content of 0.16%. Granite gravel is used as coarse aggregate, with a particle size of 4–10 mm, a density of 2.83 g/cm^3^ and a water content of 0.22%. The grain size distribution of fine aggregate and coarse aggregate according to the standard of Sand for Construction (GB/T 14684–2011) [40] and the Specification for Aggregates from Natural Sources for Concrete (BS 882:1992) [41]. The PVA fibers used in the experiment are the high-strength and high-modulus PVA short fibers produced by Kuraray Co., Ltd., (Tokyo, Japan), with a diameter of 40 μm, a length of 12 mm, a tensile strength of 1800 MPa, a dry elongation of 17%, and an alkalinity resistance of 99%.

### 2.2. Specimens and Mix Proportions

Four groups of axial compression test specimens and the corresponding 10 mixed proportions are shown in Table 2, based on the properties of materials and some try–tests. The specimens are numbered according to the fly ash mass ratio and PVA fibers volume ratios as experimental research parameters. For convenience, in this paper, the fly ash mass ratio is indicated by mt.%, while the volume ratio of PVA fibers is indicated by vol.%. For example, F40-P0.6 represents a sample with 40 mt.% fly ash and 0.6 vol.% PVA fiber. The static axial compression specimen, as shown in Figure 1 is with a diameter of 100 mm and a height of 200 mm according to ASTM C469 [42]. The process of preparing the specimen is as follows: (1) the weighed dry granite gravel, river sand, fly ash and slag were poured into the mixing pot, mixed for 8 min; (2) put PVA fibers into the mixing pot and added activator solution and water. Mixing was continued for 12 min to ensure the uniform dispersion of fibers. Note that when adding the fibers, kept the machine mixed. (3) Poured the concrete into the 100 × 200 mm molds, gently vibrated them several times, and then removed bubbles in the cement mortar vibration table; (4) demold after curing 24 h, and then covered with plastic wrap to continue curing to the target age in the room environment.

### 2.3. Test Setup

#### 2.3.1. Axial Compression Test

**Figure 2:**
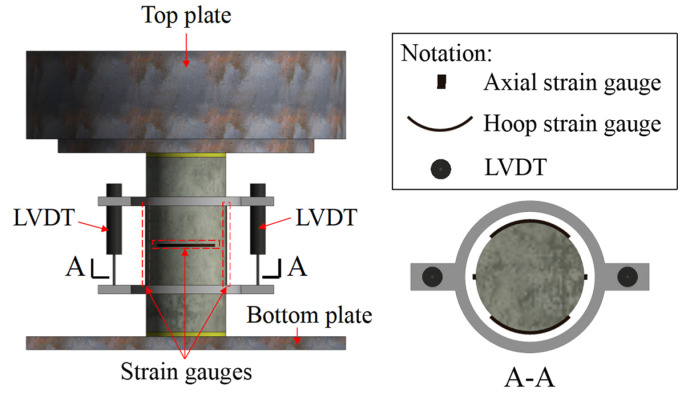
Axial compression test set-up.

Axial compression tests were carried out to verify the effect of ratios of PVA fibers and fly ash on the compression performance of the PFRGCs. The axial compression test setup is shown in Figure 2. Displacement loading mode with a rate of 0.18 mm/min is adopted on the axial compression specimens by a MATEST-5000 kN voltage servo testing machine referred to the Standard Test Methods for Fiber Reinforced Concrete (CECS 13–2009) [43] and Standard Test Mothed for Compressive Strength of Cylindrical Concrete Specimens (ASTM C39) [44]. The specimens were levelled with high-strength gypsum to ensure the axial compression load [45,46,47]. Axial strains and hoop strains of the middle height of the samples, as shown in Figure 2, were collected at a frequency of 1 Hz by the TDS-530 static acquisition instrument and measured axial deformations with two linear variable differential transformers (LVDTs) to ensure effective axial deformation measurements with the failures of strain gauges during the loading [48,49,50].

#### 2.3.2. SEM Test

**Figure 3:**
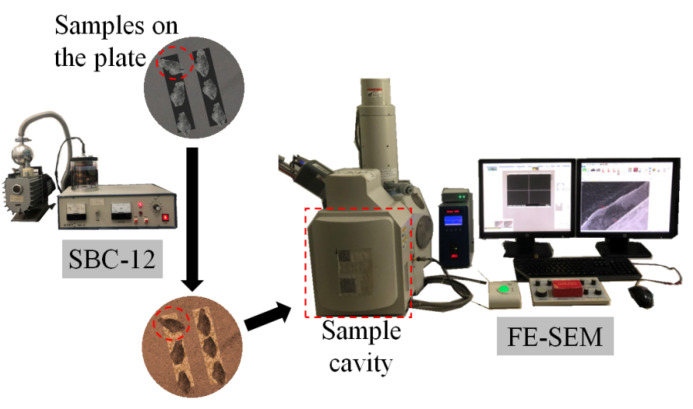
Set-up of SEM test.

To analyze the microscopic mechanism of the effect of PVA fibers and fly ash on the axial compression performance of the PFRGCs, Hitachi S-3400N were used to observe and analyze the microstructure of the damaged samples, as shown in Figure 3, as shown in Figure 3. The Hitachi S-3400N is a field emission-scanning electron microscope (FE-SEM), which can carry out high-resolution imaging and specimen morphology research from nanometer to millimeter. Specimens of the SEM test were chosen and cut from the specimens after axial tests. The specimens of SEM tests were kept dry, and gold iron was injected by an SBC-12 iron sputtering instrument before observation in the SEM cavity.

## 3. Results and Discussions

### 3.1. Failure Modes

**Figure 4:**
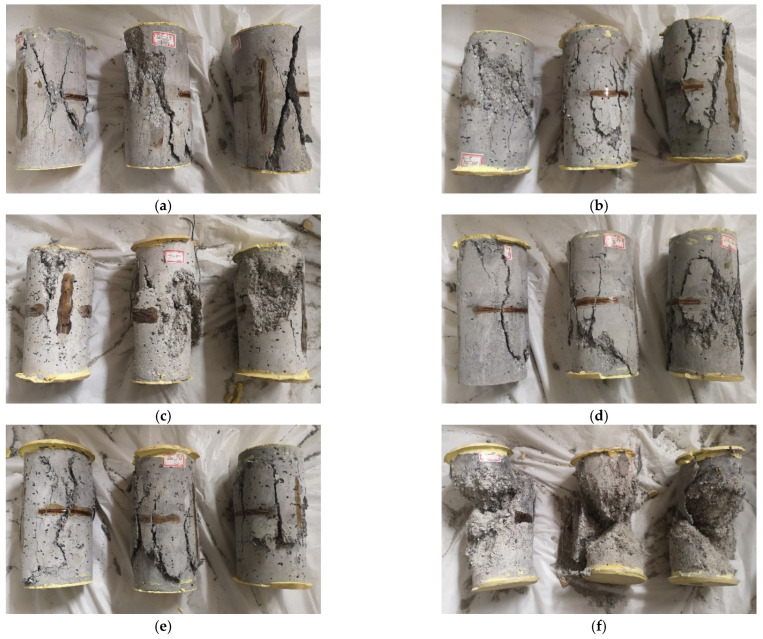

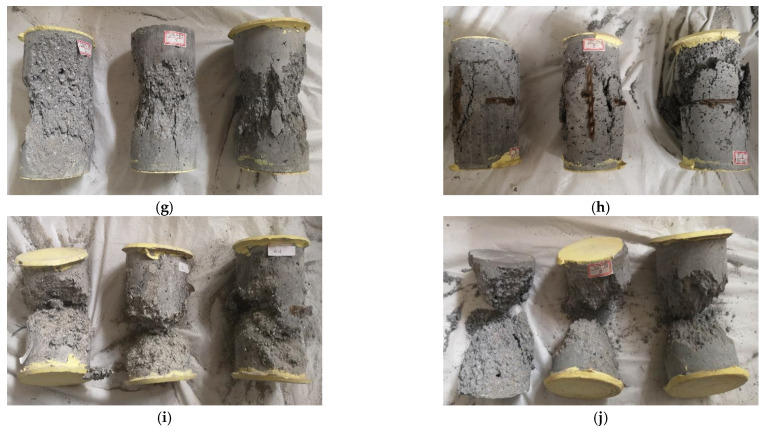
Failure modes of PFRGCs (**a**)F40-P0; (**b**) F40-P0.6; (**c**) F40-P1.2; (**d**) F60-P0; I F60-P0.6; (**f**) F60-P1.2; (**g**) F80-P0; (**h**) F80-P0.6; (**i**) F80-P1.2; (**j**) F100-P0.

Failure modes of the PFRGCs with different ratios of PVA fibers and fly ash are shown in Figure 4. The failure process of specimens without PVA fibers is divided into the following stages: (1) micro-deformation stage, micro-deformation occurred in the axial and hoop directions. Tiny micro-cracks appeared at the weak points in the concrete matrix. (2) Cracks propagation stage, with the increasing load, the micro-cracks became longer and wider, and the concrete on the surface began to collapse and peel off. (3) Specimen failure stage, the micro-cracks in the concrete grew and connected, and the micro-cracks grew into large cracks that run through the whole specimen, resulting in the overall failure of the specimen. Finally, the specimens were crushed into 2~4 pieces (Figure 4a,d,g,j), showing typical brittle failure of ordinary concrete. Unlike GCs, with adding PVA fibers, visible protrusions can be seen in the middle of the PFRGCs, and the specimens showed a multi-cracking characteristic and ductile behavior (Figure 4b,c,e,f,h,i). The loading time of specimens with PVA fibers is longer, and the development speed of surface cracks of specimens with PVA fibers is slower than that of GC. With the increase of axial load, the number of cracks increased, and the fibers in the cracks were broken or pulled out. Although the surface of the specimen is flabby, there is no peeling phenomenon, and the integrity is good. The addition of PVA fibers changed the original brittle failure mode of GCs.

It can be seen from Figure 4 that with the same fly ash mass ratio (PVA volume ratio), the number of porosities in the specimens increases with the increasing PVA fibers volume ratio (FA mass ratio). The main reasons may be as follows: (1) fluidity of cementitious material and uniformity of internal materials were deteriorating with the exceeding PVA fibers volume ratio, which decreased the axial compressive strength of the specimens; (2) the geopolymer reaction is decreased with an excessive PVA fibers ratio as the hydrophilic PVA fibers absorbed a large amount of free water during the geopolymer reaction process, which led to more porosities and cracks and lower compressive strength of the specimens.

### 3.2. Stress–Strain Curves

**Figure 5:**
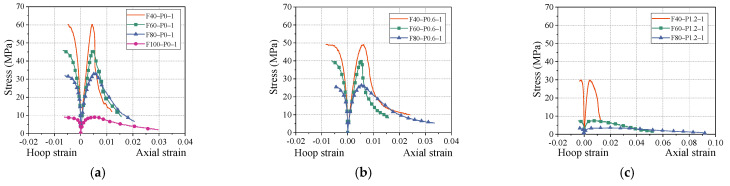
Effect of fly ash mass ratio on stress–strain relationship of PFRGCs. (**a**) 0 vol.% PVA fibers; (**b**) 0.6 vol.% PVA fibers; (**c**) 1.2 vol.% PVA fibers.

Figure 5 shows the typical axial stress–hoop strain curves and axial stress–axial strain curves of PFRGCs under different mixed proportions. The axial strains in Figure 5 were based on the average readings of two LVDTs. The specimens experienced considerable deformation in the later loading stage, which made the axial deformation monitored by SGs unreliable. For convenience, axial stress–hoop strain curves and axial stress–axial strain curves are called stress–strain curves in this paper. The axial stress–axial strain curves were terminated at around 20% of the peak load, and axial stress–hoop strain curves terminated at the peak load. As shown in Figure 5, the axial stress–axial strain of PFRGCs can be divided into four stages: (1) Linear elastic state. From the beginning of loading to about 30% of the peak stress, the micro-cracks of the specimens hardly initiate. (2) Strain softening state. The micro-cracks of the specimens begin to expand and increase, which leads to a stiffness decrease of the PFRGCs with the increasing loading. (3) Rapid decrease stage. The compressive strength of the PFRGCs rapidly decreases as the cracks expand more quickly and the crack width increase. (4) Convergence state. In this stage, due to the bridging effect of the fiber, the PFRGCs can still bear a certain compression load with large deformation. According to the stress–strain curves of the test specimens, test results including compressive strength, ultimate strain, elasticity modulus and Poisson’s ratios are shown in Table 3.

### 3.3. Compressive Strength

**Figure 6:**
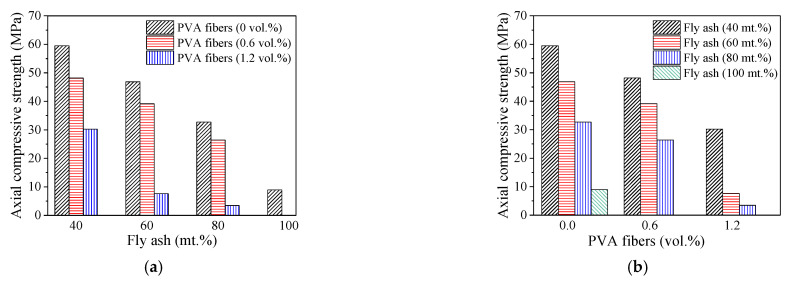
Axial compressive strength of PFRGCs with different ratios of PVA fibers/fly ash. (**a**) PFRGCs with different ratios of PVA fibers; (**b**) PFRGCs with different ratios of fly ash.

The effects of ratios of PVA fibers/fly ash on the axial compressive strength of PFRGCs with the same ratios of fly ash/PVA fibers are shown in Figure 6. The effect of PVA fibers volume ratio on the axial compressive strength of PFRGCs is shown in Figure 6a. It can be seen in Figure 6a, with the same fly ash mass ratio, the compressive strength of the specimens decreases with the increasing PVA fibers volume ratio. The compressive strength of PFRGCs with 0.6 vol.% PVA fibers is 16.5–19.8% lower than that of PFRGCs without PVA fibers (Figure 6a). With the same PVA fibers volume ratio, the influence of different fly ash mass ratios on the compressive strength of PFRGCs is shown in Figure 6b. Under the condition of three volume ratio of PVA fibers (0 vol.%, 0.6 vol.%, 1.2 vol.%), the compressive strength of PFRGCs decreases with the increasing fly ash. When the volume ratio of PVA fibers is 0 vol.% and 0.6 vol.%, the compressive strength of the specimens decreases nearly linearly with the increasing fly ash.

The influence of fly ash mass ratio on the strength of the PFRGCs is as follows: (1) the mass ratio of slag decreases with the increasing fly ash mass ratio, which increases the setting time of the concrete is prolonged; (2) smooth spherical fly ash will be dissolved by alkali solution to form C-S-H and C (N)-A-S-H. The alkali content of the solution in this test is fixed, and the number of intact fly ash particles in the concrete increases with the increasing fly ash. More crack initiation between the interface of the matrix and the undissolved fly ash particles led to the decreasing compression strength of the specimens. The influence of the PVA fiber volume ratio on the strength of PFRGCs was described above. 

### 3.4. Elastic Modulus and Poisson’s Ratio

The elastic modulus and Poisson’s ratio can be extracted from the stress–strain curves according to the ATSM standard. The equations are as follows:

(1) $$E=\frac{\sigma_{2}-\sigma_{1}}{\varepsilon_{2}-0.00005}$$

(2) $$\mu =\frac{\varepsilon_{c2}-\varepsilon_{c1}}{\varepsilon_{2}-0.00005}$$

where E is elastic modulus, $\sigma_{1}$ is the stress corresponding to the axial strain of 0.00005, $\sigma_{2}$ is 40% of the peak stress, $\varepsilon_{2}$ is the axial strain corresponding to $\sigma_{2}$, μ is Poisson’s ratio, $\varepsilon_{c2}$, $\varepsilon_{c1}$ is the hoop strain corresponding to $\sigma_{2}$ and $\sigma_{1}$, respectively.

**Figure 7:**
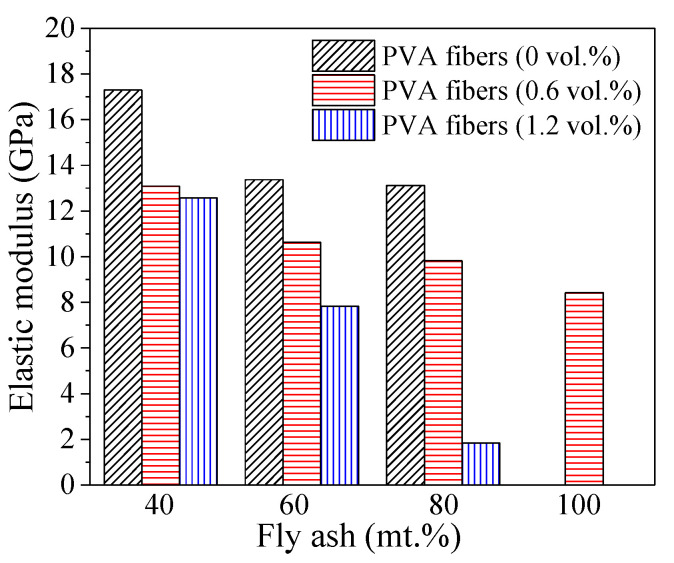
Elastic modulus of PFRGCs with different ratios of PVA fibers/fly ash.

Figure 7 shows the elastic modulus of PFRGCs. It can be observed that the elastic modulus of PFRGCs decreases with the increasing PVA fibers at a given mass ratio of fly ash. Among them, PFRGCs with 40 mt.% fly ash and 0 vol.% PVA fiber shows the highest elastic modulus of 17.3 GPa. The influence of PVA fibers on elastic modulus is mainly manifested in two aspects: (1) the incorporation of PVA fibers reduces the strength of PFRGCs, and the specific reasons have been analyzed above; (2) fibers inhibit the initiation and development of cracks, and thus improve the ductility of PFRGCs. When the fly ash mass ratio and PVA fibers volume ratios are 80% and 1.2%, respectively, the PFRGC has the lowest elastic modulus of 1.84 Gpa. As shown in Figure 7, the elastic modulus of PFRGCs decreases with the increasing fly ash at a given volume ratio of PVA fibers. The reason is that the increase of fly ash reduces the strength of PFRGCs, and the specific reasons have been analyzed above. The elastic modulus of PFRGCs with 80 mt.% fly ash is 51.4% lower than that of PFRGCs with 40 mt.% fly ash content.

**Figure 8:**
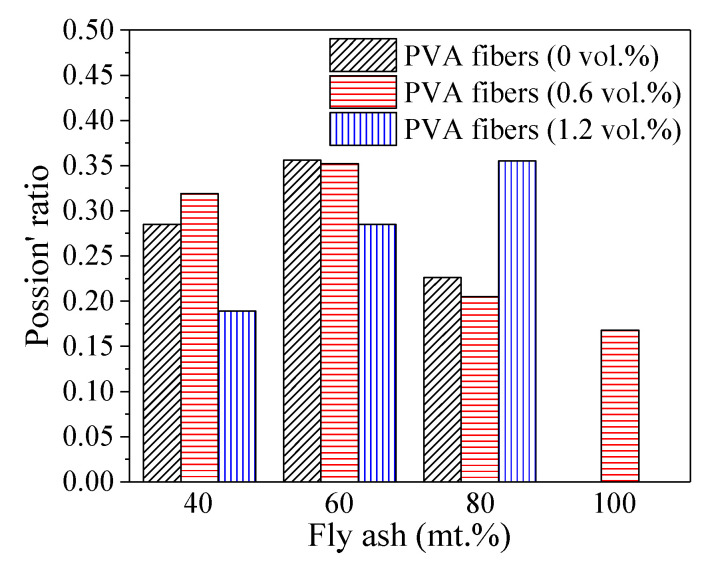
Poisson’s ratio of PFRGCs different ratios of PVA fibers/fly ash.

Figure 8 depicts Poisson’s ratio of PFRGCs under different variables. When the mass ratio of fly ash is 40% and 60%, the Poisson’s ratio of PFRGCs first increases and then decreases with the increasing PVA fibers. Many unreacted fly ash particles were doped in PFRGCs when the mass ratio of fly ash was 80%, which leads to the weak regularity of Poisson’s ratio of PFRGCs with the increasing PVA fibers. It can be observed from Figure 8 that the Poisson’s ratio of PFRGCs increases first and then decreases with the increasing fly ash when the volume ratio of PVA fibers is constant, except that the volume ratio of PVA fibers is 1.2%.

### 3.5. Microstructural Analysis Results

**Figure 9:**
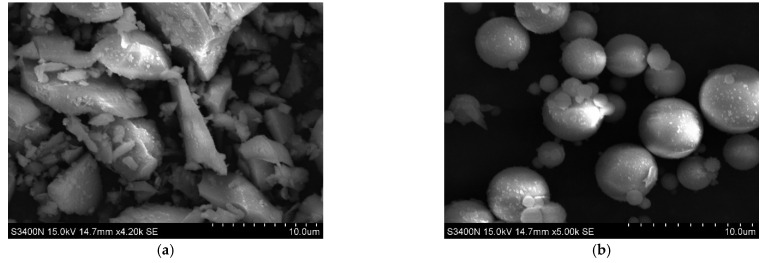
SEM photos of fly ash and slag. (**a**) Slag; (**b**) fly ash.

Figure 9 reveals the SEM photos of slag and fly ash used in this test. Slag powder is irregular flaky, or blocky (Figure 9a) while fly ash is spherical (Figure 9b). Calcium oxide accounts for a large proportion of the chemical composition of slag, so PFRGCs prepared from blast furnace slag as the primary raw material has high early strength. Because of the spherical characteristics, fly ash can improve the fluidity of concrete materials when preparing concrete, but the spherical structure has a negative impact on concrete materials.

**Figure 10:**
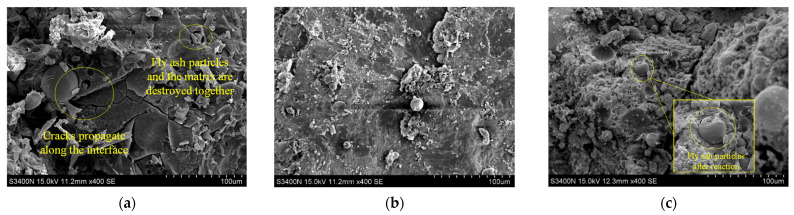
SEM photos of GCs. (**a**) F40-P0; (**b**) F60-P0; (**c**) F80-P0.

The microscopic images of GCs with different fly ash volume ratios (40 vol.%, 60 vol.% and 80 vol.%) under SEM are shown in Figure 10. It can be seen from Figure 10 that the matrix of GCs is relatively dense. Comparing the three graphs in Figure 10, the remaining spherical fly ash particles in GC increase with the increasing fly ash. There are two main failure modes of GCs: (1) the fly ash particles are well combined with the matrix, and the fly ash particles and the matrix are destroyed together; (2) the fly ash particles are poorly bonded with the matrix, and cracks propagate along with the interface between fly ash particles and the matrix.

**Figure 11:**
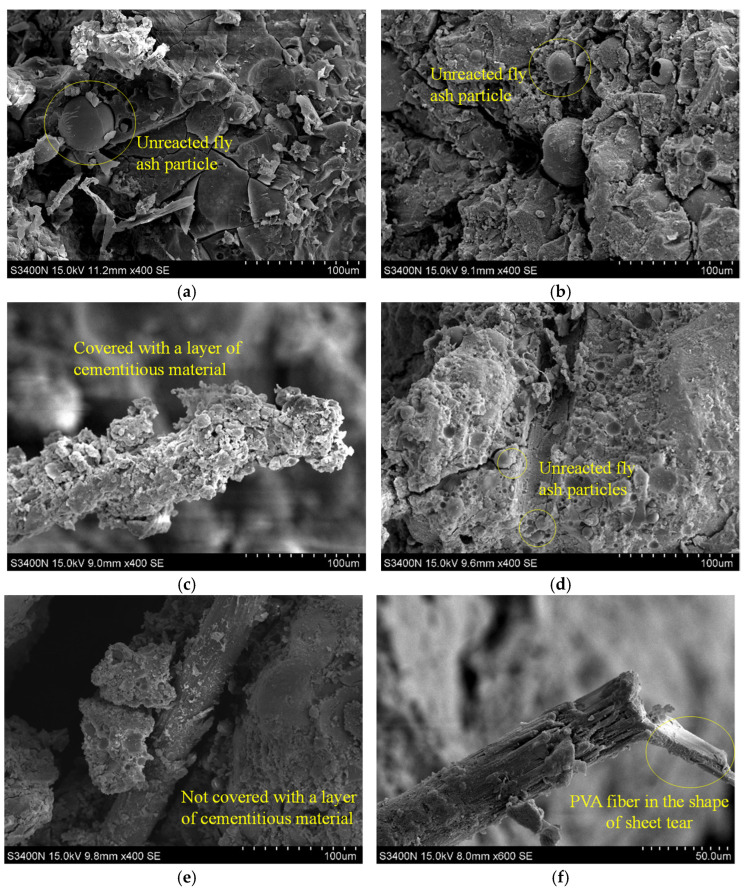
SEM photos of PFRGCs. (**a**) F40-P0; (**b**) F40-P0.6; (**c**) PVA fiber of F40-P0.6; (**d**) F40-P1.2; (**e**) PVA fiber of F40-P1.2; (**f**) Broken PVA fiber of F40-P1.2.

Figure 11 shows the SEM photos of PFRGCs with a different volume ratio of PVA fibers under the same ratio fly ash. Comparing Figure 11a,b,d, it can be known that the number of unreacted fly ash particles in PFRGCs increases with the increase of PVA fibers. This phenomenon shows that the increase of PVA fibers content affects the alkali-activated reaction. Although PVA fibers have been pulled out from the matrix of PFRGCs, the surface of PVA fibers is still covered with a layer of cementitious material (Figure 11c). Comparing the PVA fiber micrographs in Figure 11c,e, it can be found that the PVA fibers of F40-P0.6 are better combined with the matrix than of F40-P1.2. Figure 11f is a microscopic image of PVA fibers after breaking. It can be seen that the fracture of PVA fibers is in the shape of sheet tear, which indicates that the PVA fibers bridging the surface of the cracks in PFRGCs consume energy when it is pulled out and broken.

## 4. Conclusions

This paper conducted quasi-static axial compression and SEM tests on PFRGCs. The influence ratio of PVA fibers (0 vol.%, 0.6 vol.%, 1.2 vol.%) and low-calcium fly ash (40 mt.%, 60 mt.%, 80 mt.%, 100 mt.%) on compressive resistance of PFRGCs were investigated. Based on the test results and discussions, the main conclusions are as follows:

1. PFRGCs without PVA fibers (GCs) show typical brittle failure. The addition of PVA fibers improved the ductility of GCs. Different from the failure mode of GCs, visible protrusions can be seen in the middle of the PFRGCs, and the specimens showed a multi-cracking characteristic and ductile behavior.
2. The compressive strength of the specimens decreases with increasing PVA fibers at a given mass ratio of fly ash. With the increase of low-calcium fly ash/PVA fibers, the number of unreacted fly ash particles in PFRGCs increases. Too many unreacted fly ash particles make GC more prone to micro-cracks during loading, adversely affecting compressive properties. The compressive strength of PFRGCs with 0.6 vol.% PVA fibers is 16.5–19.8% lower than that of PFRGCs without PVA fibers. Under the condition of three volume ratio of PVA fibers (0 vol.%, 0.6 vol.%, 1.2 vol.%), the compressive strength of PFRGCs decreases with the increasing fly ash.
3. Under the given volume ratio of PVA fibers, the increase of fly ash leads to a decrease in compressive strength and an increase in ductility. The compressive strength of PFRGCs with 40 mt.% fly ash and without PVA fibers is the highest, while that of the PFRGCs with 80 vol.% fly ash and 1.2 mt.% PVA fibers are the weakest. With an increase in the PVA fibers at a given mass ratio of fly ash, the compressive strength of specimens decreases gradually, but the final strain has no obvious rule.
4. The elastic modulus of PFRGCs decreases with the increasing PVA fibers at a given mass ratio of fly ash. With the increase of low-calcium fly ash/PVA fibers, the number of unreacted fly ash particles in PFRGCs increases. PFRGCs with 40 mt.% fly ash and 0 vol.% PVA fibers have the highest elastic modulus of 17.3 GPa.
5. When the mass ratio of fly ash is 40% and 60%, the Poisson’s ratio of PFRGCs first increases and then decreases with the increasing PVA fibers. The Poisson’s ratio of PFRGCs increases first and then decreases with the increasing fly ash when the volume ratio of PVA fibers is constant, except that the volume ratio of PVA fibers is 1.2%.

In summary, the axial compression performance of PFRGCs was studied, which made a step forward in better understanding the influence of PVA fibers and fly ash on various physical and mechanical properties of GC materials. These properties are significant to ensure the durability and safety of structures designed with PFRGCs. However, considering the limitations of this study, further studies are needed to ensure the reliable application of PFRGCs in structural applications.

## Figures and Tables

**Table 1:** Chemical composition of low-calcium fly ash.

**Composition**	SiO_2_	Al_2_O_3_	Fe_2_O_3_	CaO	MgO	Na_2_O	SO_3_	K_2_O
**Proportion (%)**	50.8	28.1	6.2	3.7	1.2	1.2	0.8	0.6

**Table 2:** Mixing proportions.

Specimen	Mix Proportions by Weight (kg/m^3^)								Ratio (%)	
	Coarse Aggregate	Fine Aggregate	Slag	Fly Ash	Na_2_SiO_3_	NaOH	Water	PVA Fibers	Fly Ash (M_R_, %)	PVA Fibers (V_R_, %)
F40-P0	1294	554	220.80	147.20	131	53	43.20	0	40	0
F40-P0.6								14.66		0.6
F40-P1.2								29.32		1.2
F60-P0	1294	554	147.20	220.80	131	53	43.20	0	60	0
F60-P0.6								14.66		0.6
F60-P1.2								29.32		1.2
F80-P0	1294	554	73.60	294.40	131	53	43.20	0	80	0
F80-P0.6								14.66		0.6
F80-P1.2								29.32		1.2
F100-P0	1294	554	-	368.00	131	53	43.20	0	100	0

Notation: MR—Mass ratio; VR—Volume ratio.

**Table 3:** Test results of specimens under quasi-static compression loadings.

Specimens	Compressive Strength (MPa)			Ultimate Strain		Elasticity Modulus (GPa)		Poisson’s Ratios	
	Test Average			Test Average		Test Average		Test Average	
F40-P0	1	60.23	59.49	0.0047	0.0046	16.88	17.29	0.292	0.285
	2	58.25		0.0042		17.47		0.297	
	3	60.00		0.0048		17.53		0.265	
F40-P0.6	1	49.05	48.18	0.0062	0.0056	13.07	13.09	0.320	0.319
	2	47.52		0.0058		13.11		0.313	
	3	47.96		0.0046		13.08		0.324	
F40-P1.2	1	30.55	30.22	0.0038	0.0039	12.61	12.57	0.180	0.189
	2	29.88		0.0040		11.82		0.179	
	3	25.52		0.0038		13.29		0.208	
F60-P0	1	45.99	46.86	0.0047	0.0047	13.78	13.37	0.373	0.346
	2	47.52		0.0046		13.21		0.327	
	3	47.08		0.0047		13.11		0.338	
F60-P0.6	1	40.07	39.15	0.0050	0.0058	10.71	10.62	0.343	0.352
	2	38.76		0.0070		10.37		0.359	
	3	38.61		0.0051		10.79		0.355	
F60-P1.2	1	7.38	7.61	0.0042	0.0043	7.78	7.83	0.307	0.285
	2	7.72		0.0045		7.89		0.264	
	3	7.72		0.0040		7.82		0.283	
F80-P0	1	32.19	32.70	0.0047	0.0045	13.17	13.11	0.227	0.226
	2	33.06		0.0044		13.26		0.212	
	3	32.85		0.0041		12.90		0.238	
F80-P0.6	1	26.49	26.42	0.0058	0.0057	10.63	9.82	0.221	0.205
	2	24.30		0.0058		8.85		0.196	
	3	28.47		0.0054		9.97		0.198	
F80-P1.2	1	3.35	3.46	0.0036	0.0039	1.87	1.84	0.366	0.355
	2	3.69		0.0040		1.81		0.340	
	3	3.35		0.0040		1.84		0.358	
F100-P0	1	9.19	8.90	0.0035	0.0038	8.70	8.42	0.191	0.168
	2	9.19		0.0037		8.14		0.145	
	3	8.32		0.0041		8.42		0.168	

## Data Availability

Not applicable.

## References

[1] Shukla J., Verma M., Misra A. (2017). Effect of global warming on sea level rise: A modeling study. Ecol. Complex..

[2] Varotsos C., Mazei Y., Efstathiou M. (2020). Paleoecological and recent data show a steady temporal evolution of carbon dioxide and temperature. Atmos. Pollut. Res..

[3] Chuai X., Lu Q., Huang X., Gao R., Zhao R. (2021). China’s construction industry-linked economy-resources-environment flow in international trade. J. Clean. Prod..

[4] Hossain M.U., Poon C.S. (2018). Global warming potential and energy consumption of temporary works in building construction: A case study in Hong Kong. Build. Environ..

[5] Bribian I.Z., Usón A.A., Scarpellini S. (2009). Life cycle assessment in buildings: State-of-the-art and simplified LCA methodology as a complement for building certification. Build. Environ..

[6] Akan M., Övül A., Dhavale D.G., Sarkis J. (2017). Greenhouse gas emissions in the construction industry: An analysis and evaluation of a concrete supply chain. J. Clean. Prod..

[7] Peng J.X., Huang L., Zhao Y.B., Chen P., Zeng L., Zheng W. (2012). Modeling of Carbon Dioxide Measurement on Cement Plants. Adv. Mater. Res..

[8] Meyer C. (2009). The greening of the concrete industry. Cem. Concr. Compos..

[9] Jindal B.B., Jangra P., Garg A. (2020). Effects of ultra fine slag as mineral admixture on the compressive strength, water absorption and permeability of rice husk ash based geopolymer concrete. Mater. Today Proc..

[10] Lahoti M., Wong K.K., Tan K.H., Yang E.-H. (2018). Effect of alkali cation type on strength endurance of fly ash geopolymers subject to high temperature exposure. Mater. Des..

[11] Han F., Philip V. (2022). Structural performance of geopolymer-concrete-filled steel tube members subjected to compression and bending. J. Constr. Steel Res..

[12] Habert G., De Lacaillerie J.D.E., Roussel N. (2011). An environmental evaluation of geopolymer based concrete production: Reviewing current research trends. J. Clean. Prod..

[13] He J., Jie Y., Zhang J., Yu Y., Zhang G. (2013). Synthesis and characterization of redmud and rice husk ash-based geopolymer composites. Cement Concr. Compos..

[14] Zhao J., Wang K., Wang S., Wang Z., Yang Z., Shumuye E., Gong X. (2021). Effect of Elevated Temperature on Mechanical Properties of High-Volume Fly Ash-Based Geopolymer Concrete, Mortar and Paste Cured at Room Temperature. Polymer.

[15] Xiao R., Ma Y., Jiang X., Zhang M., Zhang Y., Wang Y., Huang B., He Q. (2020). Strength, microstructure, efflorescence behavior and environmental impacts of waste glass geopolymers cured at ambient temperature. J. Clean. Prod..

[16] Singh N. (2018). Fly Ash-Based Geopolymer Binder: A Future Construction Material. Minerals.

[17] Zhang H.Y., Qiu G.H., Kodur V., Yuan Z.S. (2020). Spalling behavior of metakaolin-fly ash based geopolymer concrete under elevated temperature exposure. Cem. Concr. Compos..

[18] Junaid M.T., Khennane A., Kayali O. (2014). Performance of fly ash based geopolymer concrete made using non-pelletized fly ash aggregates after exposure to high temperatures. Mater. Struct..

[19] Tittarelli F., Giosuè C., Mobili A., DI Perna C., Monosi S. (2016). Effect of Using Recycled Instead of Virgin EPS in Lightweight Mortars. Procedia Eng..

[20] Gunasekara C., Law D., Bhuiyan S., Setunge S., Ward L. (2019). Chloride induced corrosion in different fly ash based geopolymer concretes. Constr. Build. Mater..

[21] Ariffin M., Bhutta M., Hussin M., Tahir M.M., Aziah N. (2013). Sulfuric acid resistance of blended ash geopolymer concrete. Constr. Build. Mater..

[22] Castel A., Foster S.J., Ng T., Sanjayan J.G., Gilbert R.I. (2016). Creep and drying shrinkage of a blended slag and low calcium fly ash geopolymer Concrete. Mater. Struct..

[23] Sarker P., Haque R., Ramgolam K.V. (2013). Fracture behaviour of heat cured fly ash based geopolymer concrete. Mater. Des..

[24] Pan Z., Sanjayan J.G., Rangan B.V. (2011). Fracture properties of geopolymer paste and concrete. Mag. Concr. Res..

[25] Mahmood A., Noman M., Pechočiaková M., Amor N., Petrů M., Abdelkader M., Militký J., Sozcu S., Hassan S. (2021). Geopolymers and Fiber-Reinforced Concrete Composites in Civil Engineering. Polymer.

[26] de Azevedo A., Cruz A., Marvila M., de Oliveira L., Monteiro S., Vieira C., Fediuk R., Timokhin R., Vatin N., Daironas M. (2021). Natural Fibers as an Alternative to Synthetic Fibers in Reinforcement of Geopolymer Matrices: A Comparative Review. Polymer.

[27] Gülşan M.E., Alzeebaree R., Rasheed A.A., Niş A., Kurtoğlu A.E. (2019). Development of fly ash/slag based self-compacting geo-polymer concrete using nano-silica and steel fiber. Constr. Build. Mater..

[28] Bernal S., Gutierrez R.D., Delvasto S., Rodriguez E. (2010). Performance of an alkali-activated slag concrete reinforced with steel fibers. Constr. Build. Mater..

[29] Farhan N.A., Sheikh M.N., Hadi M.N.S. (2018). Engineering Properties of Ambient Cured Alkali-Activated Fly Ash–Slag Concrete Reinforced with Different Types of Steel Fiber. J. Mater. Civ. Eng..

[30] Heard W., Basu P., Slawson T., Nordendale N. (2011). Characterization and performance optimization of a cementitious composite for quasi-static and dynamic loads. Procedia Eng..

[31] Xiao B., Wang W., Zhang X., Long G., Fan J., Chen H., Deng L. (2019). A novel fractal solution for permeability and Kozeny-Carman constant of fibrous porous media made up of solid particles and porous fibers. Powder Technol..

[32] Liang M., Fu C., Xiao B., Luo L., Wang Z. (2019). A fractal study for the effective electrolyte diffusion through charged porous media. Int. J. Heat Mass Transf..

[33] Al-Mashhadani M.M., Canpolat O., Aygörmez Y., Uysal M., Erdem S. (2018). Mechanical and microstructural characterization of fiber reinforced fly ash based geopolymer composites. Constr. Build. Mater..

[34] Zhang Y.S., Sun W., Li Z.J., Zhou X.M., Eddie, Chau C.K. (2008). Impact properties of geopolymer based extrudates incorporated with fly ash and PVA short fiber. Constr. Build. Mater..

[35] Li Z., Zhang Y., Zhou X. (2005). Short Fiber Reinforced Geopolymer Composites Manufactured by Extrusion. J. Mater. Civ. Eng..

[36] Zhang Y., Sun W., Li Z., Zhou X. (2009). Geopolymer extruded composites with incorporated fly ash and polyvinyl alcohol short fiber. ACI Mater. J..

[37] Xiao S.-H., Liao S.-J., Zhong G.-Q., Guo Y.-C., Lin J.-X., Xie Z.-H., Song Y. (2021). Dynamic properties of PVA short fiber reinforced low-calcium fly ash-slag geopolymer under an SHPB impact load. J. Build. Eng..

[38] Qin L.F., Qu B., Shi C.J., Zhang J.H. (2020). Effect of Ca/Si ratio on the formation and characteristics of aluminosilicate gel. Mater. Rep..

[39] Nath P., Sarker P. (2014). Effect of GGBFS on setting, workability and early strength properties of fly ash geopolymer concrete cured in ambient condition. Constr. Build. Mater..

[40] (2011). Sand for Construction.

[41] (1992). Specification for Aggregates from Natural Sources for Concrete.

[42] (2002). Standard Test Method for Static Modulus of Elasticity and Poisson’s Ratio of Concrete in Compression.

[43] (2009). Standard Test Methods for Fiber Reinforced Concrete.

[44] (2018). Standard Test Mothed for Compressive Strength of Cylindrical Concrete Specimens.

[45] Lin J.-X., Song Y., Xie Z.-H., Guo Y.-C., Yuan B., Zeng J.-J., Wei X. (2020). Static and dynamic mechanical behavior of engineered cementitious composites with PP and PVA fibers. J. Build. Eng..

[46] Guo Y., Xie J., Zheng W., Li J. (2018). Effects of steel slag as fine aggregate on static and impact behaviours of concrete. Constr. Build. Mater..

[47] Guo Y.-C., Zhang J.-H., Chen G.-M., Xie Z.-H. (2014). Compressive behaviour of concrete structures incorporating recycled concrete aggregates, rubber crumb and reinforced with steel fibre, subjected to elevated temperatures. J. Clean. Prod..

[48] Zhou J.-K., Lin W.-K., Guo S.-X., Zeng J.-J., Bai Y.-L. (2021). Behavior of FRP-confined FRP spiral reinforced concrete square columns (FCFRCs) under axial compression. J. Build. Eng..

[49] Liao J., Yang K.Y., Zeng J.-J., Quach W.-M., Ye Y.-Y., Zhang L. (2021). Compressive behavior of FRP-confined ultra-high performance concrete (UHPC) in circular columns. Eng. Struct..

[50] Zeng J.-J., Ye Y.-Y., Quach W.-M., Lin G., Zhuge Y., Zhou J.-K. (2021). Compressive and transverse shear behaviour of novel FRP-UHPC hybrid bars. Compos. Struct..

